# Impact of carbon monoxide poisoning on the risk of breast cancer

**DOI:** 10.1038/s41598-020-77371-w

**Published:** 2020-11-24

**Authors:** Chien-Cheng Huang, Chung-Han Ho, Yi-Chen Chen, Chien-Chin Hsu, Hung-Jung Lin, Yu-Feng Tian, Jhi-Joung Wang, How-Ran Guo

**Affiliations:** 1 Department of Emergency Medicine, Chi Mei Medical Center, Tainan, Taiwan; 2 Department of Environmental and Occupational Health, College of Medicine, National Cheng Kung University, 1 Daxue Road, Tainan, 701 Taiwan; 3 Department of Senior Services, Southern Taiwan University of Science and Technology, Tainan, Taiwan; 4 Department of Medical Research, Chi Mei Medical Center, Tainan, Taiwan; 5 Department of Hospital and Health Care Administration, Chia Nan University of Pharmacy and Science, Tainan, Taiwan; 6 Department of Biotechnology, Southern Taiwan University of Science and Technology, Tainan, Taiwan; 7 Department of Emergency Medicine, Taipei Medical University, Taipei, Taiwan; 8 Division of Colorectal Surgery, Department of Surgery, Chi Mei Medical Center, Tainan, Taiwan; 9 Department of Health and Nutrition, Chia Nan University of Pharmacy and Science, Tainan, Taiwan; 10 Allied AI Biomed Center, Southern Taiwan University of Science and Technology, Tainan, Taiwan; 11 Department of Occupational and Environmental Medicine, National Cheng Kung University Hospital, Tainan, Taiwan; 12 Occupational Safety, Health and Medicine Research Center, National Cheng Kung University, Tainan, Taiwan

**Keywords:** Cancer, Environmental sciences, Diseases, Oncology

## Abstract

Carbon monoxide (CO) is a toxic gas and an endogenous signaling molecule. Some studies involving cell lines have revealed the potential antibreast cancer effects of CO. Data on such effects in humans, however, are limited. Thus, we conducted a study on patients with CO poisoning (COP) to evaluate the effects of CO on the risk of breast cancer. We identified female patients who were diagnosed with COP over the period of 2002 and 2009 from the Nationwide Poisoning Database of Taiwan. For comparison, we selected females without COP from the National Health Insurance Research Database. Participants in the COP and comparison cohorts were matched on the index year, age, monthly income, and geographic region of residence at a 1:6 ratio. We followed up the two cohorts until the end of 2014 and compared their risks of developing breast cancer. We included 7053 participants with COP and 42,318 participants without COP. Participants with COP were at a lower risk of developing breast cancer than those without COP (0.7% vs. 1.0%, *p* < 0.001). Cox proportional hazard regression analyses revealed that COP was associated with a hazard ratio of 0.67 (95% confidence interval [95% CI] 0.50–0.90) for breast cancer after we adjusted for age, monthly income, geographic region, and comorbidities of hypertension, diabetes, and hyperlipidemia. Our result provides evidence for the potential protective effects of CO against breast cancer in humans. Further studies that directly evaluate the potential effects are warranted.

## Introduction

Carbon monoxide (CO) is an exogenous toxic gas and an endogenous signaling molecule^1^. Its toxic effects are generally exerted through hypoxia and inflammation^2^. CO poisoning (COP) may increase the risk of neurologic defects^2–4^, cardiac injury^2,5^, diabetes^6^, and death^7,8^.

Endogenous CO can be produced through the action of heme oxygenases (HOs), which exist as three isoforms: HO-1, HO-2, and HO-3^9^. The abnormal metabolism and functions of endogenous CO have been linked to numerous pathologies, such as neural and cardiovascular pathologies. Similar to exogenous CO, endogenous CO functions as a physiological signaling molecule in many systems, including the neural, cardiovascular, respiratory, gastrointestinal, immune, and reproductive systems^10^. CO has antiapoptotic, anti-inflammatory, and antioxidant activities in cell and tissue homeostasis, as well as vasodilative and antiproliferative effects in tissue regeneration^10^. A growing number of studies have shown that CO has potential applications in the treatment of cancer, cardiovascular diseases, sepsis, hematological diseases, hypertension, neurodegeneration, renal diseases, and liver diseases^10^.

Breast cancer is one of the most common cancers and is a potential target disease for the therapeutic effect of CO^1^. CO might introduce protective effects against breast cancer through suppressing heat shock protein (HSP) 90^11^. Synthesized manganese carbonyl complex, a CO-releasing molecule (CORM), exerts toxic effects on breast cancer cells^12^. However, studies on the potential antibreast cancer effect of CO have been limited to cell and animal experiments. Therefore, we conducted an epidemiological study to investigate the potential effect of CO on the risk of breast cancer in human beings.

## Material and methods

### Data sources

We conducted this nationwide population-based cohort study by using the Taiwan National Health Insurance Research Database (NHIRD). The NHIRD, which covers nearly 100% of the population in Taiwan, is maintained by the National Health Research Institute and is provided to scientists for research purposes^13^.

### Study design, setting, and participants

**Figure 1:**
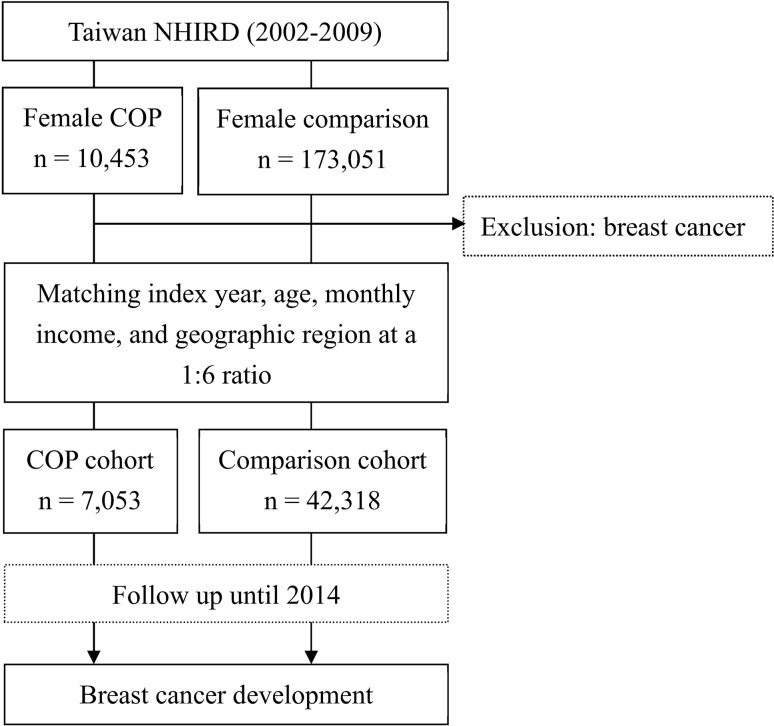
Flowchart of the present study. *NHIRD* National Health Insurance Research Database, *COP* carbon monoxide poisoning.

We identified female patients who were diagnosed with COP over the period of 2002 and 2009 as the study cohort. We selected a comparison cohort of females without COP from the NHIRD. The two cohorts were matched at a 1:6 ratio by index year, age, monthly income, and geographic region. The index year was defined as the year of hospitalization or visit to the emergency department by the patient with COP (Fig. 1). As a national policy, the Taiwanese government provides free mammogram screening once every 2 years to women aged 40–44 years who have one first-degree relative with breast cancer and all women aged 45–69 years^14^.

### Variable definitions

We defined a patient with COP as a participant who was assigned diagnosis codes 986, E868, E952, or E982 in accordance with the International Classification of Diseases, Ninth Revision, Clinical Modification (ICD-9-CM) during hospitalization or a visit to the emergency department. A patient with breast cancer was defined as a participant who had been assigned ICD-9-CM diagnosis codes 174 or 175 during at least one hospitalization or at least three visits for ambulatory care. Those who were diagnosed with breast cancer before the index date were excluded from the study.

We categorized the participants into five groups on the basis of age: < 20, 20–34, 35–49, 50–64, and ≥ 65 years^3^. Common underlying comorbidities, including hypertension (ICD-9-CM 401–405), diabetes (ICD-9-CM 250), and hyperlipidemia (ICD-9-CM 272), were included for analyses. A patient with these diseases was defined as a participant who had been assigned the relevant codes during at least one hospitalization or at least three visits for ambulatory care before the index date^3^. We categorized the participants into three groups in accordance with monthly income: < 20,000, 20,000–40,000, and > 40,000 New Taiwan Dollars (NTD)^3^.

### Statistical methods

We followed up the two cohorts until 2014 to compare their breast cancer risks. We applied independent *t*-tests to evaluate differences in continuous variables and χ^2^ tests to evaluate those in categorical variables. Cox proportional hazard regression with competing risk analysis was used to identify the independent predictors of breast cancer and evaluate their effects. We also performed multivariate regression to adjust for potential confounding effects. In addition, we applied the Kaplan–Meier method and the log-rank test to compare the breast cancer risks of the two cohorts during the follow-up period. Given that the risk of COP-associated breast cancer might change over time, we conducted further analyses by using a cutoff of 1 year for follow-up duration. All analyses were performed by using SAS 9.4 for Windows (SAS Institute, Cary, NC, USA) at a two-tailed significance level of 0.05.

### Ethical approval and consent to participate

The study protocol was reviewed and approved by the Institutional Review Board (IRB) at the Chi Mei Medical Center. Informed consent from the participants was waived by the IRB because the NHIRD contains anonymized information only. The waiver did not affect the rights and welfare of the participants.

## Results

We included 7053 female patients with COP and 42,318 females without COP in this study (Fig. 1, Table 1). In the COP cohort, the mean age was 34.1 years (standard deviation = 14.4 years), and the 20–34-year-old group had the highest number of participants (3650, 51.8%), followed by the 35–49-year-old group (2552, 35.8%). No differences in the distributions of age, monthly income, geographic region or the prevalence of hypertension, diabetes, or hyperlipidemia existed between the two cohorts. The COP cohort had a lower risk of breast cancer than the comparison cohort (0.7% vs. 1.0%, *p* < 0.001). The average age at diagnosis of breast cancer was similar between COP and comparison cohorts (50.0 vs. 49.2 years old, *p* = 0.569).

**Table 1:** Age, comorbidities, monthly income, and geographic area of residence of COP and comparison cohorts.

Variable	COP cohort (n = 7053)	Comparison cohort (n = 42,318)	*p* value
Age (years)	34.1 ± 14.4	33.9 ± 14.6	0.272
**Age subgroup (years)**			
20–34	3650 (51.8)	21,904 (51.8)	0.999
35–49	2522 (35.8)	15,136 (35.8)	
50–64	703 (10.0)	4222 (10.0)	
≥ 65	178 (2.5)	1056 (2.5)	
**Comorbidity**			
Hypertension	193 (2.7)	1145 (2.7)	0.883
Diabetes	37 (0.5)	208 (0.5)	0.714
Hyperlipidemia	28 (0.4)	168 (0.4)	> 0.999
**Monthly income (NTD)**			
< 20,000	3475 (49.3)	20,865 (49.3)	0.997
20,000–40,000	2533 (35.9)	15,198 (35.9)	
> 40,000	1045 (14.8)	6255 (14.8)	
**Geographic region**			
North	3955 (56.1)	23,734 (56.1)	> 0.999
Center	1300 (18.4)	7802 (18.4)	
South	1687 (23.9)	10,114 (23.9)	
East	111 (1.6)	668 (1.6)	
**Competing event**			
Breast Cancer	48 (0.7)	426 (1.0)	< 0.001
Mortality	629 (8.9)	799 (1.9)	

*COP* carbon monoxide poisoning, *NTD* New Taiwan dollars.

Data are expressed as mean ± standard deviation or n (%).

**Figure 2:**
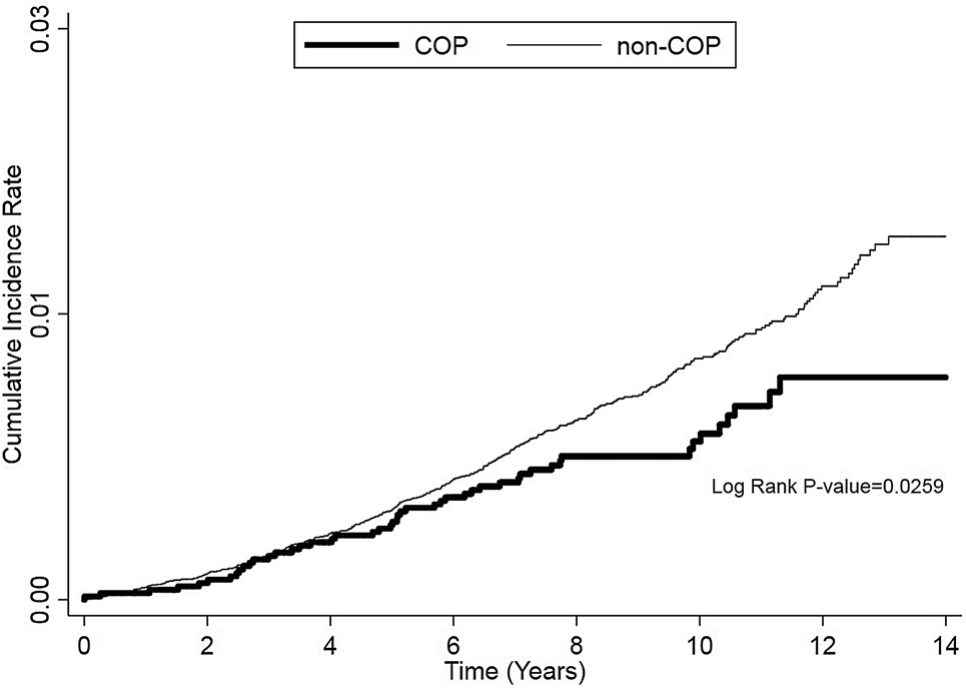
Comparison between the breast cancer risks of the COP and non-COP (comparison) cohorts during follow-up. The comparison was performed through the Kaplan–Meier’s method and the log-rank test. COP, carbon monoxide poisoning.

Cox proportional hazard regression with competing risk analysis showed that COP was associated with a hazard ratio (HR) of 0.67 (95% confidence interval [CI] 0.50–0.91; *p* = 0.009) (Table 2). The decrease in the risk associated with COP persisted after we adjusted for age, monthly income; geographic region; and hypertension, diabetes, and hyperlipidemia comorbidities (adjusted HR [AHR]: 0.67; 95% CI 0.50–0.90; *p* = 0.009). The Kaplan–Meier’s method and log-rank test also showed that the COP cohort had a lower breast cancer risk than the comparison cohort (Fig. 2).

**Table 2:** Independent predictors for breast cancer in all patients of the two cohorts during the overall follow-up period.

Variable	Crude model HR^SD^ (95% CI)	*p* value	Full model HR^SD^ (95% CI)*	*p* value
**Cohort**				
Comparison	1 (reference)		1 (reference)	
COP	0.67 (0.50–0.91)	0.009	0.67 (0.50–0.90)	0.009
**Age (years)**				
20–34	1 (reference)		1 (reference)	
35–49	4.72 (3.70–6.01)	< 0.001	4.67 (3.66–5.96)	< 0.001
50–64	6.60 (4.96–8.78)	< 0.001	6.03 (4.49–8.10)	< 0.001
≥ 65	2.96 (1.62–5.42)	< 0.001	2.17 (1.07–4.39)	0.031
**Comorbidity**				
Hypertension	2.68 (1.87–3.84)	< 0.001	1.78 (1.16–2.73)	0.008
Diabetes	1.70 (0.64–4.53)	0.292	0.87 (0.29–2.67)	0.813
Hyperlipidemia	2.82 (1.17–6.81)	0.021	1.28 (0.46–3.52)	0.637
**Monthly income (NTD)**				
< 20,000	0.80 (0.62–1.02)	0.070	0.75 (0.59–0.97)	0.029
20,000–40,000	0.77 (0.60–1.01)	0.057	0.79 (0.61–1.03)	0.087
> 40,000	1 (reference)		1 (reference)	
**Geographic region**				
North	1 (reference)		1 (reference)	
Center	0.65 (0.50–0.85)	0.002	0.76 (0.58–0.99)	0.045
South	0.84 (0.68–1.05)	0.126	0.91 (0.72–1.13)	0.385
East	0.55 (0.23–1.34)	0.190	0.67 (0.28–1.64)	0.384

The independent predictors were identified through competing risk regression analysis.

*COP* carbon monoxide poisoning, *HR* hazard ratio, *AHR* adjusted hazard ratio, *HR*^*SD*^ adjusted competing risks hazard ratio, *CI* confidence interval, *NTD* New Taiwan Dollars.

*Adjusted for age, hypertension, diabetes, hyperlipidemia, monthly income, and geographic region.

In further analyses stratified by follow-up duration, we found that COP was associated with a HR of 0.52 (95% CI 0.12–2.21; *p* = 0.378) in the first year of follow-up (Table 3). Moreover, the AHR remained the same as the HR after we adjusted for age; monthly income; geographic region; and hypertension, diabetes, and hyperlipidemia comorbidities (0.52, 95% CI 0.12–2.21; *p* = 0.377). The reduction in risk was larger than that observed over the whole follow-up period but did not reach statistical significance. COP was associated with a HR of 0.70 (95% CI 0.52–0.96; *p* = 0.024) (Table 4) after 1 year of follow-up, and the AHR was close to the HR after adjustment for other variables (0.71, 95% CI 0.52–0.96; *p* = 0.026). We divided COP cohort into those with one and multiple episodes COPs and compared them with the comparison cohort. The result showed that both those with one and multiple episodes of COP had a lower risk for breast cancer than the comparison cohort (0.70% and 0.30%, respectively). However, the difference between those with one and multiple episodes of COP did not reach statistical significance (Supplementary Table S1). The Kaplan–Meier’s method and log-rank test also showed that the COP cohort was at a lower risk for breast cancer than the comparison cohort in the first year of follow-up (see Supplementary Fig. S1) and after 1 year of follow-up (see Supplementary Fig. S2).

**Table 3:** Independent predictors for breast cancer in all patients of the two cohorts in the first year of follow-up.

Variable	Crude model HR^SD^ (95% CI)	*p* value	Full model HR^SD^ (95% CI)*	*p* value
**Cohort**				
Comparison	1 (reference)		1 (reference)	
COP	0.52 (0.12–2.21)	0.378	0.52 (0.12–2.21)	0.377
**Age (years)**				
20–34	1 (reference)		1 (reference)	
35–49	3.98 (1.27–12.50)	0.018	3.70 (1.12–12.22)	0.032
50–64	11.68 (3.60–37.93)	< 0.001	8.56 (2.49–29.48)	< 0.001
≥ 65	5.18 (0.58–46.38)	0.141	1.87 (0.09–37.46)	0.683
**Comorbidity**				
Hypertension	6.85 (2.35–19.94)	< 0.001	2.87 (0.66–12.46)	0.161
Diabetes	8.35 (1.14–61.50)	0.037	2.10 (0.16–27.30)	0.571
Hyperlipidemia	10.50 (1.42–77.49)	0.024	1.87 (0.10–36.86)	0.679
**Monthly income (NTD)**				
< 20,000	0.44 (0.10–1.96)	0.282	0.50 (0.11–2.27)	0.369
20,000–40,000	0.61 (0.13–2.86)	0.528	0.66 (0.13–3.23)	0.607
> 40,000	1 (reference)		1 (reference)	
**Geographic region**				
North	1 (reference)		1 (reference)	
Center	0.61 (0.18–2.10)	0.432	0.66 (0.19–2.32)	0.522
South	1.10 (0.45–2.68)	0.843	1.06 (0.43–2.62)	0.900
East	–	–	–	–

The independent predictors were identified through competing risk regression analysis.

*COP* carbon monoxide poisoning, *HR* hazard ratio, *AHR* adjusted hazard ratio, *HR*^*SD*^ adjusted competing risks hazard ratio, *CI* confidence interval, *NTD* New Taiwan Dollars.

*Adjusted for age, hypertension, diabetes, hyperlipidemia, monthly income, and geographic region.

**Table 4:** Independent predictors for breast cancer in all patients of the two cohorts after 1 year of follow-up.

Variable	Crude model HR^SD^ (95% CI)	*p* value	Full model HR^SD^ (95% CI)*	*p* value
**Cohort**				
Comparison	1 (reference)		1 (reference)	
COP	0.70 (0.52–0.96)	0.024	0.71 (0.52–0.96)	0.026
**Age (years)**				
20–34	1 (reference)		1 (reference)	
35–49	4.77 (3.72–6.11)	< 0.001	4.74 (3.69–6.08)	< 0.001
50–64	6.39 (4.76–8.59)	< 0.001	5.93 (4.37–8.04)	< 0.001
≥ 65	3.02 (1.61–5.66)	< 0.001	2.34 (1.14–4.77)	0.020
**Comorbidity**				
Hypertension	2.55 (1.74–3.74)	< 0.001	1.71 (1.10–2.65)	0.017
Diabetes	1.43 (0.46–4.41)	0.537	0.76 (0.22–2.66)	0.661
Hyperlipidemia	0.24 (0.91–6.47)	0.078	1.16 (0.39–3.43)	0.789
**Monthly income (NTD)**				
< 20,000	0.77 (0.60–0.98)	0.037	0.73 (0.56–0.94)	0.015
20,000–40,000	0.76 (0.58–0.99)	0.040	0.78 (0.60–1.02)	0.069
> 40,000	1 (reference)		1 (reference)	
**Geographic region**				
North	1 (reference)		1 (reference)	
Center	0.65 (0.50–0.86)	0.003	0.76 (0.58*–*1.01)	0.057
South	0.83 (0.66–1.04)	0.112	0.90 (0.71–1.14)	0.376
East	0.58 (0.24–1.41)	0.230	0.71 (0.29–1.74)	0.455

The independent predictors were identified through competing risk regression analysis.

*COP* carbon monoxide poisoning, *HR* hazard ratio, *AHR* adjusted hazard ratio, *HR*^*SD*^ adjusted competing risks hazard ratio, *CI* confidence interval, *NTD* New Taiwan Dollars.

*Adjusted for age, hypertension, diabetes, hyperlipidemia, monthly income, and geographic region.

## Discussion

This study revealed that female patients with COP had a lower risk for breast cancer than those without COP. This epidemiologic study provided an interesting finding and indirect evidence for the potential role of CO in breast cancer treatment.

The reduction in breast cancer risk after CO exposure may be attributed to the possible direct toxic effect of CO and to the death of cancer cells due to severe hypoxia. Vítek et al. used CO gas (500 ppm 1 h/day) to treat mice that had been xeno transplanted subcutaneously with pancreatic cancer cells^15^. They found that CO exposure significantly inhibits the proliferation of human pancreatic cancer cells and doubles the survival rates of mice^15^. Nemeth et al. reported that low doses of CO block lung cancer progression by modulating myeloid cell/macrophage infiltration and phenotype in the tumor microenvironment^16^. The induction of apoptosis in lung tumors is associated with the increased expression of CD86 and the activation of mitogen-activated protein kinase/extracellular signal-regulated kinases 1/2 pathway^16^. Grau et al. found that CO inhalation could increase tumor hypoxia, which may affect tumor control^17^. Whereas hypoxia may play a role in tumor progression^18^, an acute episode of severe hypoxia may lead to the death of cancer cells before the resistance to hypoxic environment being developed. Therefore, we may reasonably speculate that CO exposure before the clinical diagnosis of breast cancer might affect precancerous cells or existing cancer cells in patients with COP.

Studies on endogenous CO may cast some light on the anticancer effect of CO. Endogenous CO is a byproduct of the oxidative conversion of heme^1^. The conversion of heme to biliverdin, ferrous iron, and CO is catalyzed by HO-1 and HO-2^1^. Biliverdin is further reduced to bilirubin by biliverdin reductase^1^. The role of HO-3 is not fully understood. HO-3 is suspected to be a pseudogene that is derived from HO-2 transcripts^19^. HO-1 is found in the spleen, liver, vascular endothelial cells, and smooth muscle tissues^1^. It is the only inducible HO isoform, and its increase is stimulated by cellular stress^1^. HO-2 is responsible for neurotransmission and vascular tone regulation^9,20^. HO-2 and HO-3 are ubiquitously expressed in the brain, liver, and testes^9,20^. CO maintains cell and tissue homeostasis via its antiapoptotic, anti-inflammatory, and antioxidant effects^1^. CO also has antiproliferative and vasodilative effects and may participate in tissue regeneration and in strengthening the innate immune system^1,10^.

Although CO may have therapeutic applications, its possible toxicity stemming from its effect on oxygen transport and toxic dose control from systemic CO gas administration are major concerns^1^. COP may contribute to hypoxia and inflammation and subsequently to neurologic and cardiac dysfunction, injury to other organs, and even death^2–8^. In recent years, CORMs, a group of transition metal carbonyls or boranocarbonates that can release CO upon transformation, have provided another avenue for CO application^1,9,10,20^. High CO concentrations exert cytotoxic effects via the inhibition of the mitochondrial respiratory system, the induction of oxidative stress, and the production of reactive oxygen species^1,21^. CORMs may enable the localized release of high amounts of CO for specific cytotoxicity against targeted tumors^1^.

A growing number of studies have revealed that CO administration is an emerging hope for cancer treatment. Lee et al. found that treatment with RuCO, a type of CORM, reduced the growth of human MCF7 and MDA-MB-231 breast cancer cells^11^. RuCO down-regulated the expression of growth-related proteins, including cyclinD1, CDK4, and hTERT^11^. Given that HSP90 stabilizes several proteins required for tumor growth, the feasibility of using HSP90 inhibitors as anticancer drugs has been investigated^22^. The contradictory effects of RuCO treatment on wild-type and mutant p53 proteins are similar to those of cells treated with geldanamycin, a HSP90 inhibitor; this similarity suggests that RuCO might affect HSP90 activity^11^. Fac-[MnBr(azpy)(CO)_3_], a manganese carbonyl complexes which is a photo-CORMs that release CO after irradiation with low-power visible light, was found to eradicate breast cancer cells in a dose-dependent manner and kill nearly 40% of breast cancer cells at the concentration of 75 μM^12^. In recent years, an increasing number of novel CORMs have shown potential as anticancer treatments. These CORMs include [Fe^II^(CO)(N_4_Py)](ClO_4_)_2_ for prostate cancer^23^; [Mn(CO)_3_(tpm)]PF_6_ for colon cancer^24^; fac-[MnBr(azpy)(CO)_3_] for cervical cancer, in addition to breast cancer^12^; and CORM-2 for pancreatic cancer^15^, skin cancer^25^, lymphoma, and acute myeloid leukemia^26^. In addition to CDRMs, CO gas also showed anticancer potential against pancreatic cancer^15^ and lung adenocarcinoma^27^.

The major strength of the present study is its nationwide population-based design with a large sample. While the novel finding of a decreased risk for breast cancer in COP patients has implicated the potential use of CO as a therapeutic agent, this study has several limitations. First, information on several risk factors, including family history, reproductive history, physical activity, and body mass index, is not available in the NHIRD. Consequently, we were unable to adjust for the effects of these potential confounders. Second, we did not recruit male participants given the rare incidence of breast cancer in the male population. Therefore, the results of this study might not be applied to the male population. Third, the participants were relatively young (about 34 years old on average at the beginning of follow-up). However, nearly half of patients (48.2%) were aged ≥ 35 years initially, and the peak age at the diagnosis of breast cancer in Taiwanese women was 45 to 50 years^28^. Therefore, we believe following up the participants for 12 years in the present study is sufficient to cover the age at the highest risk for a substantial portion of them. Fourth, we did not evaluate the survival advantage associated with COP or compare the distributions of breast cancer stage and ER/PR/HER2 status between patients with and without COP because they are out of the scope of the present study and the databases do not contain the some of the information. Separate studies are needed to clarify these issues. Fifth, the number of breast cancers was relatively small due to the relatively young age at the beginning of follow-up. Nonetheless, the size of patients was large enough to provide sufficient statistical power to detect the effect of COP on breast cancer. As to the potential antitumor effect, recruiting more patients and further animal and laboratory studies are needed to support its clinical application.

## Conclusions

This study demonstrated that female patients with COP had a lower risk of breast cancer than those without COP. This result may be attributed to the direct and indirect inhibitory effects of CO on tumor growth. Further studies involving collection of complete variables for possible confounders in the patients with breast cancer as well as animal and laboratory trial experiments on the mechanisms are warranted.

## Supplementary information

Supplementary Legends.

Supplementary Table S1.

Supplementary Figure S1.

Supplementary Figure S2.

## References

[1] Kourti M, Jiang WG, Cai J (2017). Aspects of carbon monoxide in form of CO-releasing molecules used in cancer treatment: more light on the way. Oxid. Med. Cell Longev..

[2] Weaver LK (2009). Clinical practice. Carbon monoxide poisoning. N. Engl. J. Med..

[3] Huang C-C (2018). Impact of hyperbaric oxygen therapy on subsequent neurological sequelae following carbon monoxide poisoning. J. Clin. Med..

[4] Huang C-C (2017). Demographic and clinical characteristics of carbon monoxide poisoning: nationwide data between 1999 and 2012 in Taiwan. Scand. J. Trauma Resusc. Emerg. Med..

[5] Huang C-C (2019). Risk of myocardial infarction after carbon monoxide poisoning: a nationwide population-based cohort study. Cardiovasc. Toxicol..

[6] Huang C-C (2017). Increased risk for diabetes mellitus in patients with carbon monoxide poisoning. Oncotarget.

[7] Huang C-C (2017). Hyperbaric oxygen therapy is associated with lower short- and long-term mortality in patients with carbon monoxide poisoning. Chest.

[8] Huang C-C (2014). Long-term prognosis of patients with carbon monoxide poisoning: a nationwide cohort study. PLoS ONE.

[9] Zobi F (2013). CO and CO-releasing molecules in medicinal chemistry. Future Med. Chem..

[10] Gullotta F, di Masi A, Ascenzi P (2012). Carbon monoxide: an unusual drug. IUBMB Life.

[11] Lee WY (2014). The induction of heme oxygenase-1 suppresses heat shock protein 90 and the proliferation of human breast cancer cells through its byproduct carbon monoxide. Toxicol. Appl. Pharmacol..

[12] Carrington SJ, Chakraborty I, Mascharak PK (2013). Rapid CO release from a Mn(I) carbonyl complex derived from azopyridine upon exposure to visible light and its phototoxicity toward malignant cells. Chem. Commun. (Camb.).

[13] Database, N. H. R. *Background*. https://nhird.nhri.org.tw/en/index.html. Accessed 30 Jan 2020.

[14] Wang W-L, Hsu S-D, Wang J-H, Huang L-C, Hsu W-L (2014). Survey of breast cancer mammography screening behaviors in Eastern Taiwan based on a health belief model. Kaohsiung J. Med. Sci..

[15] Vitek L (2014). Antiproliferative effects of carbon monoxide on pancreatic cancer. Dig. Liver Dis..

[16] Nemeth Z (2016). Alterations of tumor microenvironment by carbon monoxide impedes lung cancer growth. Oncotarget.

[17] Grau C, Khalil AA, Nordsmark M, Horsman MR, Overgaard J (1994). The relationship between carbon monoxide breathing, tumour oxygenation and local tumour control in the C3H mammary carcinoma in vivo. Br. J. Cancer.

[18] Michiels C (2004). Physiological and pathological responses to hypoxia. Am. J. Pathol..

[19] Hayashi S (2004). Characterization of rat heme oxygenase-3 gene. Implication of processed pseudogenes derived from heme oxygenase-2 gene. Gene.

[20] Foresti R, Bani-Hani MG, Motterlini R (2008). Use of carbon monoxide as a therapeutic agent: promises and challenges. Intens. Care Med..

[21] Lo Iacono L (2011). A carbon monoxide-releasing molecule (CORM-3) uncouples mitochondrial respiration and modulates the production of reactive oxygen species. Free Radic. Biol. Med..

[22] Csermely P, Schnaider T, Soti C, Prohaszka Z, Nardai G (1998). The 90-kDa molecular chaperone family: structure, function, and clinical applications. A comprehensive review. Pharmacol. Ther..

[23] Jackson CS, Schmitt S, Dou QP, Kodanko JJ (2011). Synthesis, characterization, and reactivity of the stable iron carbonyl complex [Fe(CO)(N4Py)](ClO4)2: photoactivated carbon monoxide release, growth inhibitory activity, and peptide ligation. Inorg. Chem..

[24] Niesel J (2008). Photoinduced CO release, cellular uptake and cytotoxicity of a tris(pyrazolyl)methane (tpm) manganese tricarbonyl complex. Chem. Commun. (Camb.).

[25] Allanson M, Reeve VE (2007). Carbon monoxide signalling reduces photocarcinogenesis in the hairless mouse. Cancer Immunol. Immunother..

[26] Loureiro A (2015). Folic acid-tagged protein nanoemulsions loaded with CORM-2 enhance the survival of mice bearing subcutaneous A20 lymphoma tumors. Nanomedicine.

[27] Loboda A, Jozkowicz A, Dulak J (2015). HO-1/CO system in tumor growth, angiogenesis and metabolism—targeting HO-1 as an anti-tumor therapy. Vasc. Pharmacol..

[28] Health Promotion Administration, M. O. H. A. W. *Cancer Registry Annual Report, 2017 Taiwan* (2019). https://www.hpa.gov.tw/Pages/Detail.aspx?nodeid=269&pid=12235. Accessed 17 Sept 2020.

