# Supplementary material for: Impact of carbon monoxide poisoning on the risk of breast cancer
Source: Sci Rep. 2020 Nov 24;10:20450. doi: 10.1038/s41598-020-77371-w (PMC7687884; doi:10.1038/s41598-020-77371-w)
Supplement: Supplementary file 1 — Supplementary Legends. [file 41598_2020_77371_MOESM1_ESM.docx]

**Impact of carbon monoxide poisoning on the risk of breast cancer**

Chien-Cheng Huang, MD, PhD^1-3^, Chung-Han Ho, PhD^4,5^, Yi-Chen Chen, MS^4^, Chien-Chin Hsu, MD, PhD^1,6^, Hung-Jung Lin, MD, MBA^1,6,7^, Yu-Feng Tian, MD^8,9^, Jhi-Joung Wang, MD, PhD^4,10^, How-Ran Guo, MD, MPH, ScD^2,11,12^

^1^Department of Emergency Medicine, Chi Mei Medical Center, Tainan, Taiwan

^2^Department of Environmental and Occupational Health, College of Medicine, National Cheng Kung University, Tainan, Taiwan

^3^Department of Senior Services, Southern Taiwan University of Science and Technology, Tainan, Taiwan

^4^Department of Medical Research, Chi Mei Medical Center, Tainan, Taiwan

^5^Department of Hospital and Health Care Administration, Chia Nan University of Pharmacy and Science, Tainan, Taiwan

^6^Department of Biotechnology, Southern Taiwan University of Science and Technology, Tainan, Taiwan

^7^Department of Emergency Medicine, Taipei Medical University, Taipei, Taiwan

^8^Division of Colorectal Surgery, Department of Surgery, Chi Mei Medical Center, Tainan, Taiwan

^9^Department of Health and Nutrition, Chia Nan University of Pharmacy and Science, Tainan, Taiwan

^10^Allied AI Biomed Center, Southern Taiwan University of Science and Technology, Tainan, Taiwan

^11^Department of Occupational and Environmental Medicine, National Cheng Kung University Hospital, Tainan, Taiwan

^12^Occupational Safety, Health and Medicine Research Center, National Cheng Kung University Hospital, Tainan, Taiwan

**Running title:** Carbon monoxide poisoning and breast cancer

**Corresponding Author:**

**How-Ran Guo*,*** MD, MPH, ScD, Department of Environmental and Occupational Health, College of Medicine, National Cheng Kung University, 1 Daxue Road, Tainan 701, Taiwan.

Tel: +886-6-235-3535; Fax: +886-6-275-2484; Email: hrguo@mail.ncku.edu.tw

Number of Tables: 4

Number of Figures: 2

Number of Supplementary Table: 1

Number of Supplementary Figures: 2

**Word counts:** Abstract: 239; the full article: 2,383

**Supplementary material**

**Supplementary Figure 1**–Comparison between the breast cancer risks of the COP and non-COP cohorts during the first year of follow-up. The comparison was performed through Kaplan–Meier’s method and the log-rank test. COP, carbon monoxide poisoning.

**Supplementary Figure 2**–Comparison between the breast cancer risks of the COP and non-COP cohorts after 1 year of follow-up. The comparison was performed through Kaplan–Meier’s method and the log-rank test. COP, carbon monoxide poisoning.
