# Supplementary material for: Impact of carbon monoxide poisoning on the risk of breast cancer
Source: Sci Rep. 2020 Nov 24;10:20450. doi: 10.1038/s41598-020-77371-w (PMC7687884; doi:10.1038/s41598-020-77371-w)
Supplement: Supplementary file 2 — Supplementary Table S1. [file 41598_2020_77371_MOESM2_ESM.docx]

**Supplementary Table 1.** Comparison for the incidence of breast cancer between comparison cohort and one COP cohort and between comparison cohort and multiple COPs cohort

|  | Comparison  N=42,318 | One COP  N=6,717 | *p*-value* | Multiple COPs  N=336 | *p*-value* |
| --- | --- | --- | --- | --- | --- |
| Breast cancer | 426 (1.01%) | 47 (0.70%) | 0.015 | 1 (0.30%) | 0.272 |

*p-value was estimated using Fisher’s exact test and compared with comparison cohort
