# Supplementary figures and images for: Impact of carbon monoxide poisoning on the risk of breast cancer
Source: Sci Rep. 2020 Nov 24;10:20450. doi: 10.1038/s41598-020-77371-w (PMC7687884; doi:10.1038/s41598-020-77371-w)

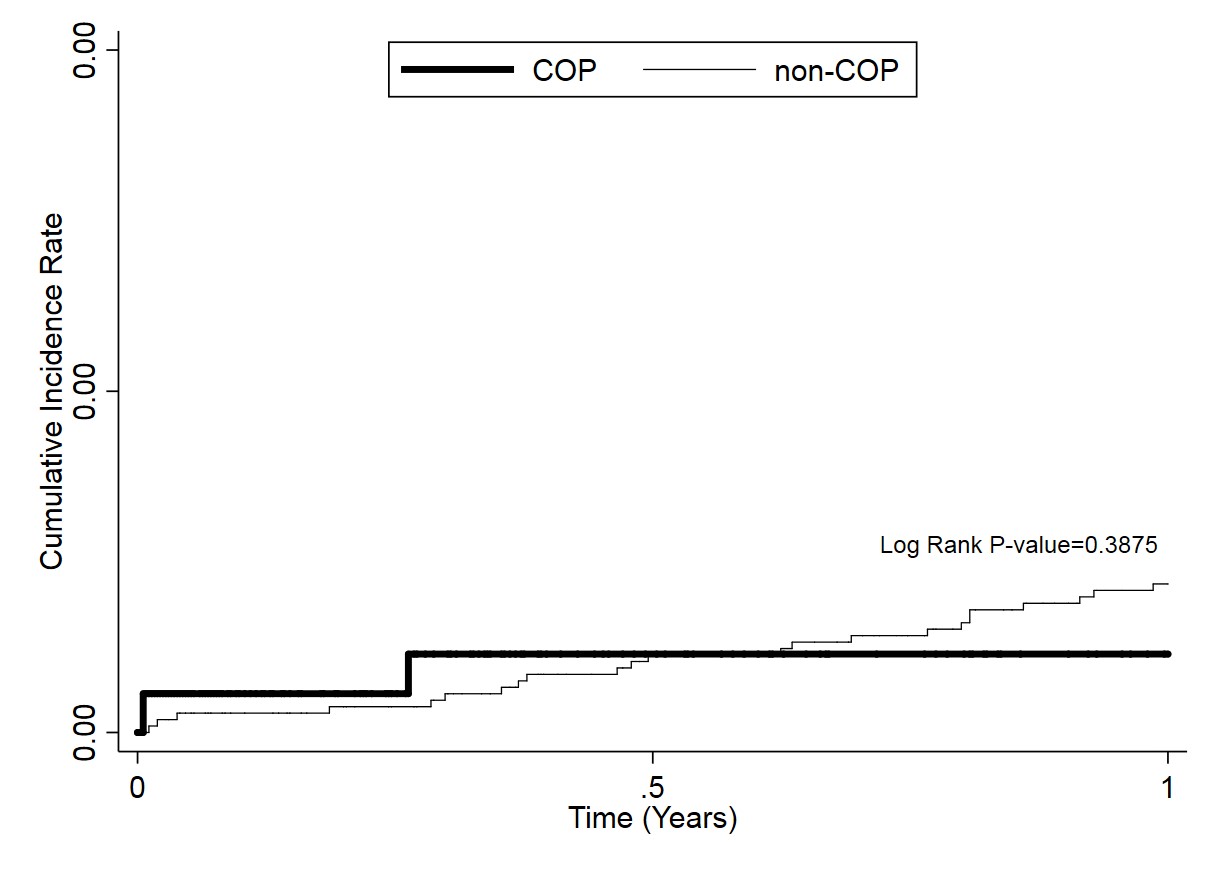

Supplement: Supplementary file 3 — Supplementary Figure S1. [file 41598_2020_77371_MOESM3_ESM.jpg]

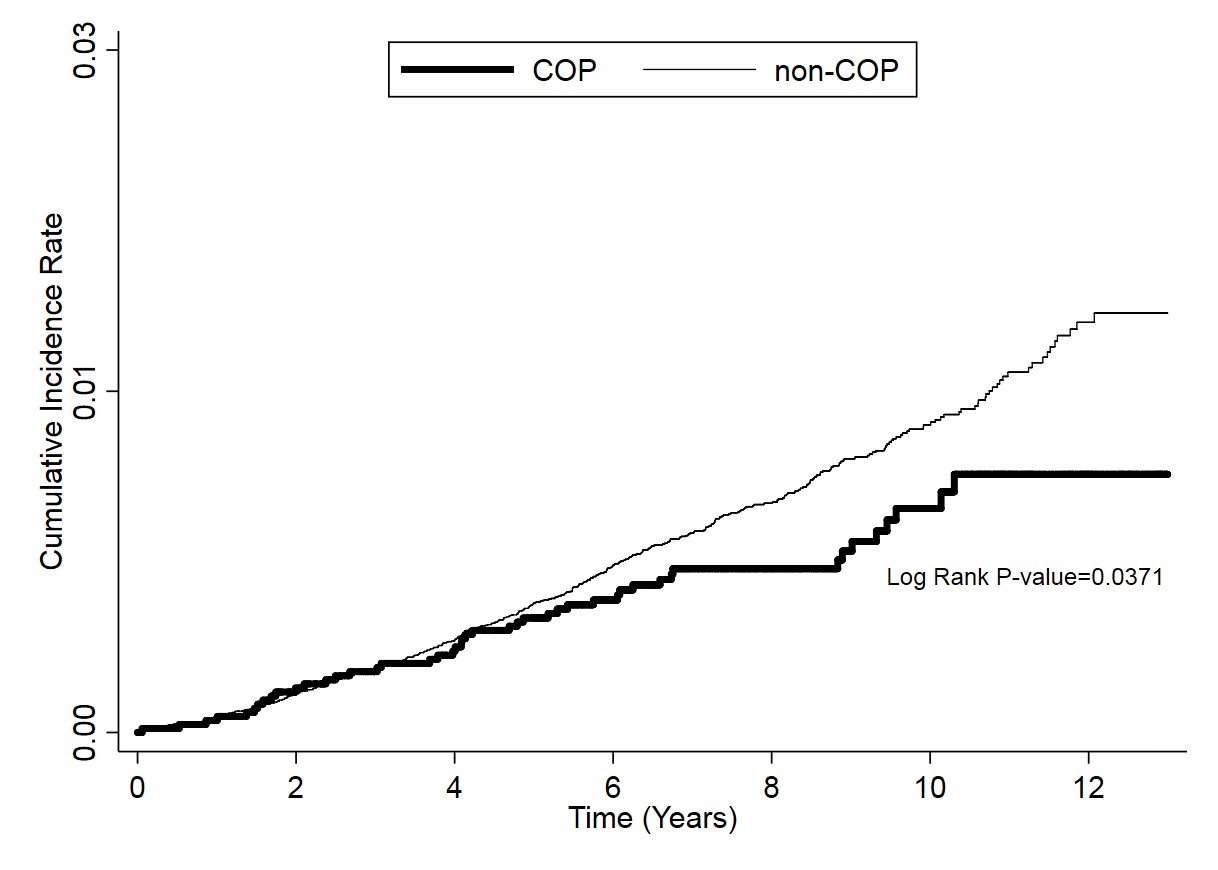

Supplement: Supplementary file 4 — Supplementary Figure S2. [file 41598_2020_77371_MOESM4_ESM.jpg]
